# Supplementary material for: GSK-J4 Inhibition of KDM6B Histone Demethylase Blocks Adhesion of Mantle Cell Lymphoma Cells to Stromal Cells by Modulating NF-κB Signaling
Source: Cells. 2023 Aug 6;12(15):2010. doi: 10.3390/cells12152010 (PMC10416905; doi:10.3390/cells12152010)
Supplement: Supplementary file 1 [file cells-12-02010-s001.zip › cells-2499985-supplementary.pdf]

# GSK-J4 inhibition of KDM6B histone demethylase blocks adhesion of Mantle Cell Lymphoma cells to stromal cells by modulating NF-κB signaling

***Laia Sadeghi [1*], Anthony P. Wright[1#]***

[1] *Division of Biomolecular and Cellular Medicine, Department of Laboratory Medicine, Karolinska Institutet, 17177 Stockholm, Sweden*

[*] *Correspondence: (laia.sadeghi@ki.se)*

[#] anthony.wright@ki.se

**This file includes:**

Supplementary text
Figures S1 to S9 (not allowed for Brief Reports)
Tables S1 to S3 (not allowed for Brief Reports)

## Figure S1.

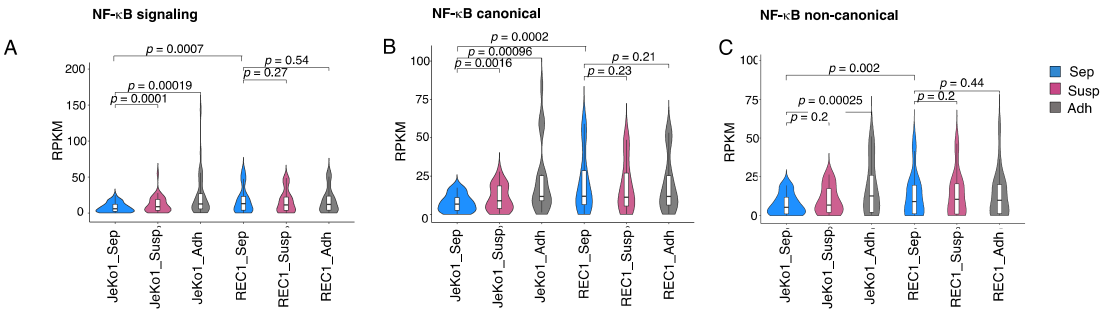


**Figure S1. Adhesion of MCL cells to stromal cells is associated with and requires induced NF-κB activity**

Violin plot showing the distribution of mRNA (FPKM values, reads per kilobase per million) of all genes required for activation of NF-κB signaling pathway listed in Table S1 and reference 41 (A), canonical NF-κB (B) and non-canonical NF-κB (C) in mono-cultured (Sep in blue) and co-cultured (Suspension: Susp in magenta and Adherent: Adh in gray) JeKo-1 and REC-1 cells. Box edges represent the interquartile range. P-values are calculated using Wilcoxon pair-wised test.

**Figure S2.**

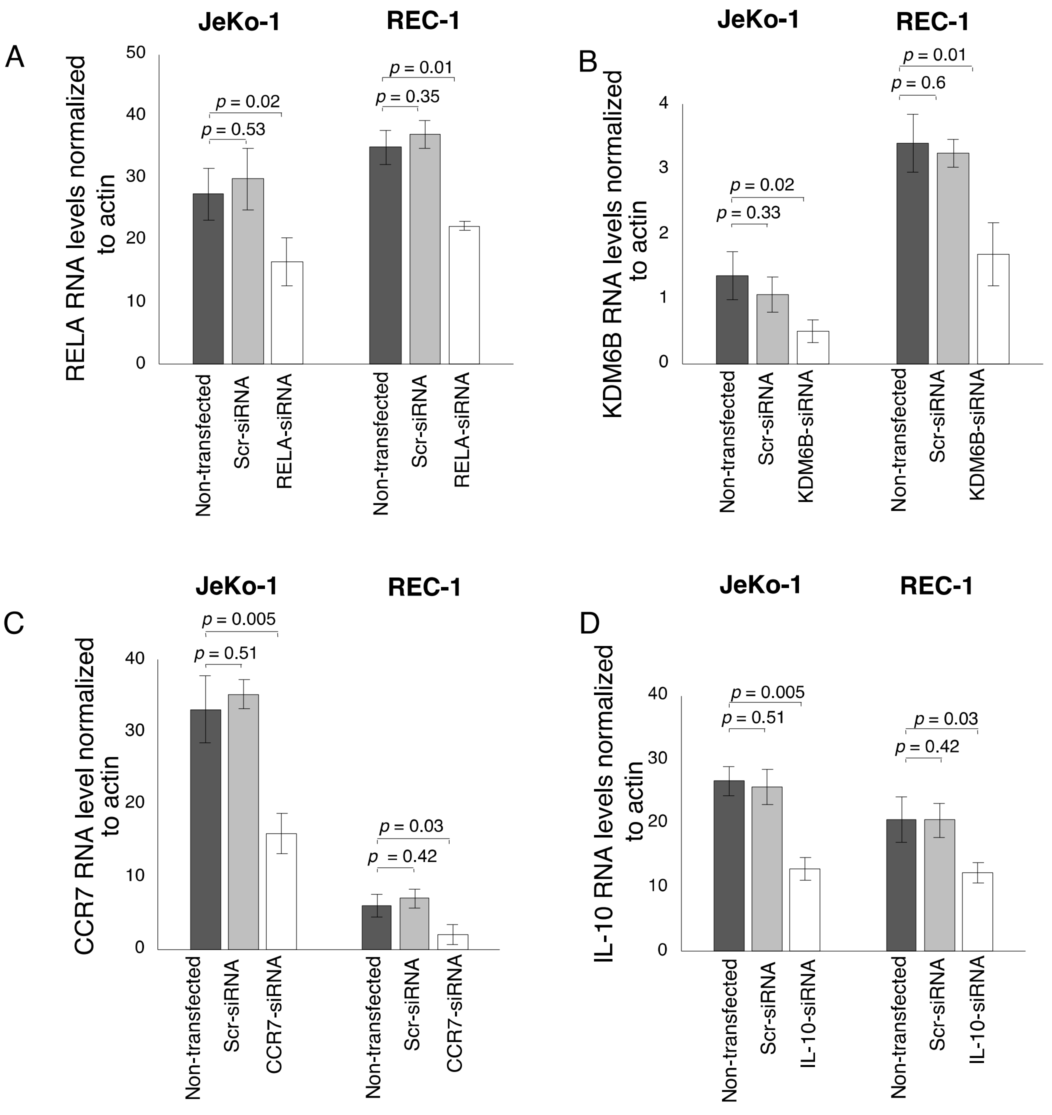


**Figure S2. siRNA-mediated knockdown of RELA, KDM6B, CCR7 and IL-10 in MCL cells**

Apparent knockdown of A) RELA, B) KDM6B, C) CCR7 and D) IL-10 measured by quantitative PCR in JeKo-1 and REC-1 cells 24 h after transfection. Error bar represents standard deviation of 3 independent experiments. Student's t-test was performed, and the *P*-values indicate the significance differences between transfected and non-transfected cells.

**Figure S3**

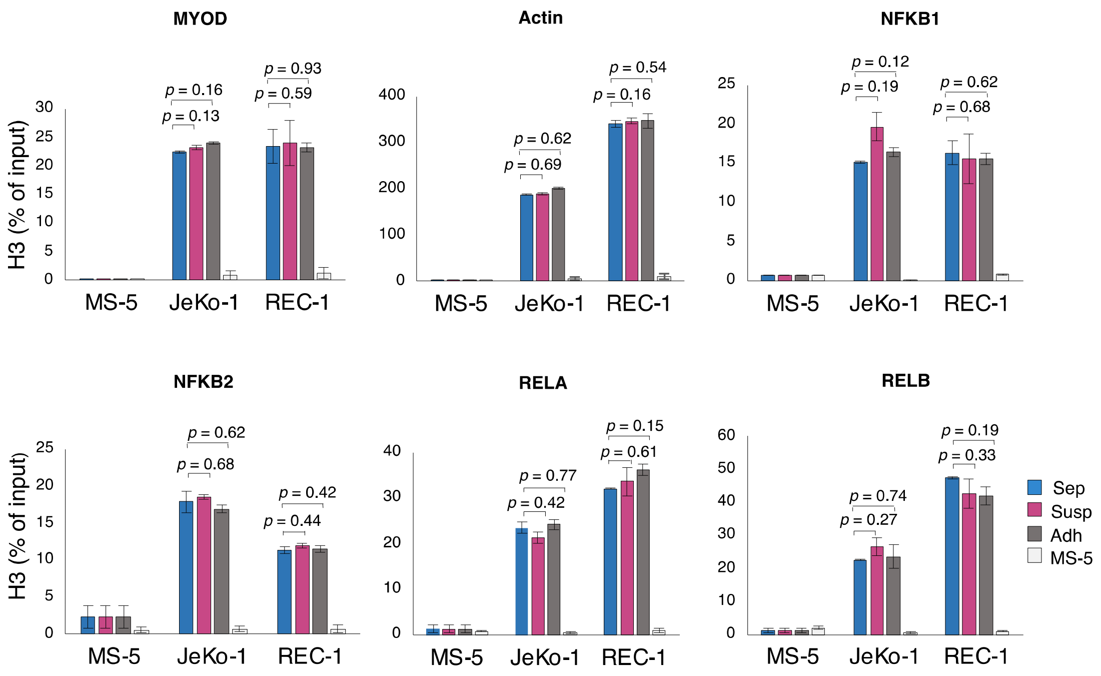


**Figure S3. Histone H3 levels at the promoter region of NF-κB gens in MCL cells**

Quantitative PCR analysis of chipped DNA (ChIP-qPCR) performed against histone H3 for promoter region of NF-κB genes in mono- (Sep, blue) and co-cultured (Suspension, Susp in magenta and adhered, Adh in gray) JeKo-1 and REC-1 cells. MS-5 stromal cells used as control to quantify primers specificity against human genome. Data were normalized to the percent of input for each sample. Error bar represents standard deviation of 3 independent experiments. Student's t-test was performed, and the *P*-values indicate the significance differences between mono- and co-cultured cells.

**Figure S4.**

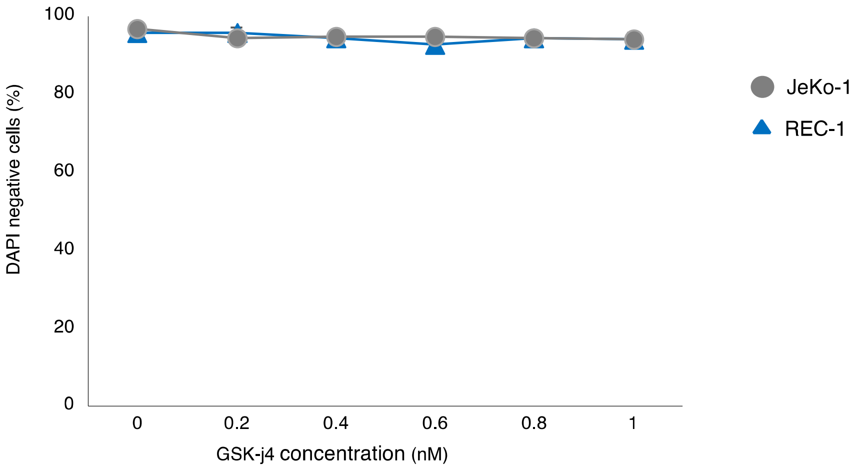


**Figure S4. Effect of GSK-J4 on MCL cells viability**

Suspension fraction of MCL cells in co-culture were stained with DAPI and the number of DAPI negative cells were calculated as percentage of total events.

**Figure S5.**

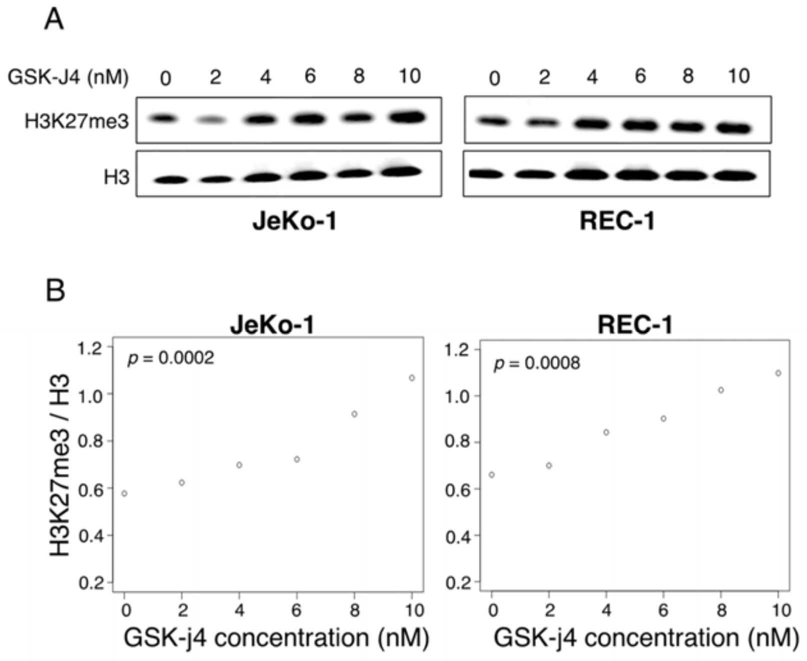


**Figure S5. Inhibition of KDM6B using GSK-J4 increases nuclear H3K27me3 levels in MCL cells**

A) Histone H3K27me3 and H3 levels in nuclear fraction of JeKo-1 and REC-1 cells after 24 h of treatment with different concentrations of GSK-J4 (0, 2, 4, 6, 8, 10 nM) is shown by western blot analysis. B) Scatter blot showing the relationship between KDM6B inhibitor (GSK-J4) and H3K27me3 levels in nuclear fraction of JeKo-1 and REC-1 cells.

**Figure S6**

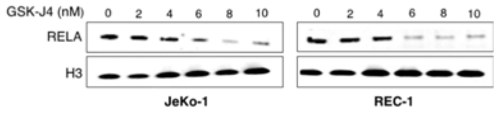


**Figure S6. Inhibition of KDM6B using GSK-J4 decreased nuclear RELA levels in MCL cells**
RELA and H3 levels in nuclear fraction of JeKo-1 and REC-1 cells after 24 h of treatment with different concentrations of GSK-J4 (0, 2, 4, 6, 8, 10 nM) is shown by western blot analysis.

**Figure S7**

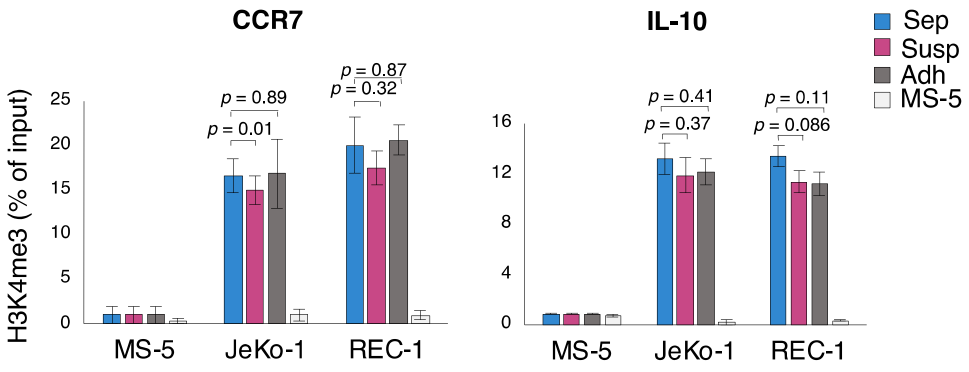


**Figure S7. H3K4me3 levels at the promoter region of CCR7 and IL10 in MCL cells**

Quantitative PCR analysis of chipped DNA (ChIP-qPCR) performed against H3K4me3 for promoter region of NF-κB target genes (CCR7 and IL-10) in mono- (Sep, blue) and co-cultured (Suspension, Susp in magenta and adhered, Adh in gray) JeKo-1 and REC-1 cells. Data were normalized to the percent of input for each sample. Error bar represents standard deviation of 3 independent experiments. Student's t-test was performed, and the *P*-values indicate the significance differences between mono- and co-cultured cells.

**Figure S8**

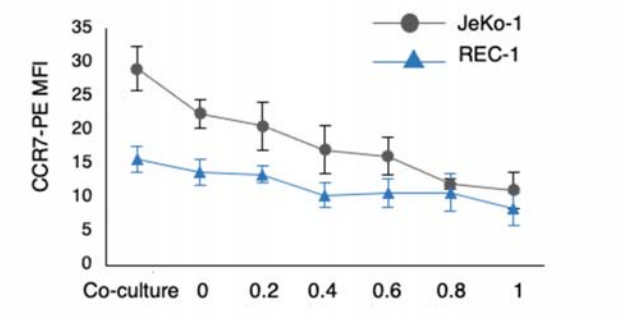


**Figure S8. CCR7 cell surface levels in MCL cells after GSK-J4 treatment**

Membrane expression level of CCR7 in co-cultured and mono-cultured JeKo-1 and REC-1 cells without and with different concentration of GSK-J4 (0, 0.2, 0.4, 0.6, 0.8 and 1 nM) was quantified using flow cytometry and presented as mean fluorescent intensity (*n* = 3).

**Figure S9**

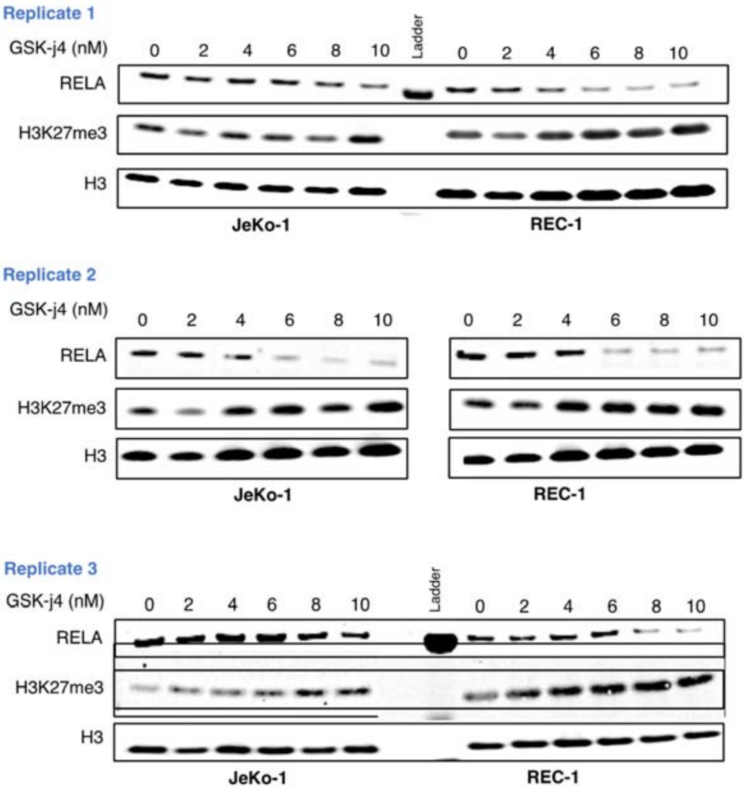


**Figure S9. H3K27me3 and RELA protein levels from 3 independent experiments**

Histone H3K27me3, H3 and RELA levels in nuclear fraction of JeKo-1 and REC-1 cells after 24 h of treatment with different concentrations of GSK-J4 (0, 2, 4, 6, 8, 10 nM) is shown by western blot analysis from 3 different experiments shown in Figure. 4A and supplemental Figure. S4B

**Table S1. NF-κB signaling pathway associated genes. All genes regulate NF-κB signaling pathway, 1: indicate genes involved in canonical or non-canonical pathways. FPKM values are taken from (41).**

| ensemble | name | canonical | non-canonical |
|---|---|---|---|
| ENSG00000109320 | NFKB1 | 1 | 0 |
| ENSG00000173039 | RELA | 1 | 0 |
| ENSG00000162924 | REL | 1 | 0 |
| ENSG00000167604 | NFKBID | 1 | 0 |
| ENSG00000100906 | NFKBIA | 1 | 0 |
| ENSG00000104825 | NFKBIB | 1 | 0 |
| ENSG00000167604 | NFKBID | 1 | 0 |
| ENSG00000232810 | TNF | 1 | 0 |
| ENSG00000172936 | MYD88 | 1 | 0 |
| ENSG00000198001 | IRAK4 | 1 | 0 |
| ENSG00000144802 | NFKBIZ | 1 | 0 |
| ENSG00000136869 | TLR4 | 1 | 0 |
| ENSG00000213341 | CHUK | 1 | 0 |
| ENSG00000104365 | IKBKB | 1 | 0 |
| ENSG00000269335 | IKBKG | 1 | 0 |
| ENSG00000142867 | BCL10 | 1 | 0 |
| ENSG00000172175 | MALT1 | 1 | 0 |
| ENSG00000028137 | TNFRSF1B | 1 | 0 |
| ENSG00000067182 | TNFRSF1A | 1 | 0 |
| ENSG00000277632 | CCL3 | 1 | 0 |
| ENSG00000110330 | BIRC2 | 0 | 1 |
| ENSG00000023445 | BIRC3 | 0 | 1 |
| ENSG00000056558 | TRAF1 | 0 | 1 |
| ENSG00000127191 | TRAF2 | 0 | 1 |
| ENSG00000131323 | TRAF3 | 0 | 1 |
| ENSG00000082512 | TRAF5 | 0 | 1 |
| ENSG00000175104 | TRAF6 | 0 | 1 |
| ENSG00000006062 | MAP3K14 | 0 | 1 |
| ENSG00000077150 | NFKB2 | 0 | 1 |
| ENSG00000104856 | RELB | 0 | 1 |
| ENSG00000101017 | CD40 | 0 | 1 |
| ENSG00000226979 | LTA | 0 | 1 |
| ENSG00000227507 | LTB | 0 | 1 |
| ENSG00000121594 | CD80 | 0 | 1 |
| ENSG00000069399 | BCL3 | 0 | 1 |
| ENSG00000139193 | CD27 | 0 | 1 |
| ENSG00000178562 | CD28 | 0 | 1 |
| ENSG00000090339 | ICAM1 | 0 | 1 |

**Table S2. List of primers used for gene expression in the study**

| Gene | Sequence |
|---|---|
| RELB-F | AAAACGTCTCGGGGGTAATC |
| RELB-R | CGAAGCCGTTCTCCTTGAT |
| NFKB1-F | TTGGCTTAACGTTCACCTTTG |
| NFKB1-R | GGCACCAGGTAGTCCACCAT |
| RELA-F | ACCTGAGGAGAGCAAGTCCC |
| RELA-R | GTTTCTCCTCAATCCGGTGAC |
| NFKB2-F | TTCCCGATCTGAGTCCAGGT |
| NFKB2-R | GCTTGTCTCGGGTTTCTGGA |
| Actin-F | GGCGGCTGAGTTTGTTTACG |
| Actin-R | TGGCGAAGGATTTCCCCATC |
| KDM6B-F | CACCCCAGCAAACCATATTATGC |
| KDM6B-R | CACACAGCCATGCAGGGATT |
| CCR7-F | TGAGGTCACGGACGATTACAT |
| CCR7-R | GTAGGCCCACGAAACAAATGAT |
| IL10-F | ACTTACACAGCGCCGTAGCC |
| IL10-R | CCTTGTCTGAGATGATCCAG |

**Table S3. List of primers used for ChIP-seq in the study**

| Gene | Sequence |
|---|---|
| MYOD-F | CCGCCTGAGCAAAGTAAATGA |
| MYOD-R | GGCAACCGCTGGTTTGG |
| RELB-F | CCTGCCAACCTCTCGATCCTGAAGC |
| RELB-R | CACGCGTCACGTGATCGCGGGGGCGG |
| NFKB1-F | GACACATCCGGACCTCGCAGGGGCTAGTCGG |
| NFKB1-R | GTTTGTAAGAGTTCCCCTCCGGTTCGCGTTC |
| RELA-F | CGATACAGCCAAACTGCCGGCTCTCATC |
| RELA-R | CACAAGCGCCAGGCTTCAGAGACGAGGCTCC |
| NFKB2-F | GATACGCGGACCCGTACGTACACCTG |
| NFKB2-R | GGACACGGGTGAGCGTATCTTGAGTTTGTG |
| CCR7-F | CCCCACTATCTCTGGTCTTGG |
| CCR7-R | GAGTCCTCTGAATGAACCTTC |
| IL10-F | CCCAGTCAGGAGGACCAGGCAAC |
| IL10-R | CTACACATCAGGGGCTTGCTCTTGC |
